# Human analysts or artificial intelligence? Source attribution in athlete performance evaluation

**DOI:** 10.3389/fpsyg.2026.1954191

**Published:** 2026-09-10

**Authors:** Jun-Phil Uhm, Minkyu Kim, Soojung Park, Minjeong Kwak, Seunghyeon Moon, Kwonhyeon Kang

**Affiliations:** 1 Department of Kinesiology, Inha University, Incheon, Republic of Korea; 2 Department of Multicultural Education, Inha University, Incheon, Republic of Korea

**Keywords:** artificial intelligence, individualized consideration, message credibility, perceived diagnosticity, source attribution, sport analytics, task-dependent algorithm aversion

## Abstract

**Introduction:**

Artificial intelligence (AI) is increasingly used to support athlete performance evaluation, yet responses to AI-based analysis may depend on whether the evaluative task is perceived as requiring objective calculation or contextual human judgment. Drawing on task-dependent algorithm aversion, this study examined whether an identical athlete performance report would be evaluated differently when attributed to an AI-based analysis system or professional human analysts.

**Methods:**

A total of 220 participants were randomly assigned to review the same football performance report presented with either an AI-based system or professional human analysts as its source. Participants evaluated the report’s message credibility, perceived diagnosticity, and consideration of the athlete’s unique characteristics. A one-way multivariate analysis of covariance and follow-up univariate analyses were conducted while controlling for familiarity with the athlete.

**Results:**

Source attribution had a significant multivariate effect on the combined outcomes. The AI-attributed report received significantly higher evaluations of message credibility and perceived diagnosticity, whereas the human-attributed report was perceived as giving greater consideration to the athlete’s unique characteristics.

**Discussion:**

The findings imply that algorithm appreciation and algorithm aversion can coexist within the same evaluative task. AI attribution was associated with more favorable evaluations on dimensions linked to systematic information processing, whereas human attribution was associated with greater perceived individualized and context-sensitive understanding.

## 1 Introduction

Artificial intelligence (AI) is increasingly being applied to the measurement, prediction, and interpretation of athlete performance (Li et al., 2025). Machine-learning techniques have been used to predict performance and injury risk in team sports, while deep-learning and computer-vision methods have expanded the automated recognition of sport-specific movements (Chmait and Westerbeek, 2021; Claudino et al., 2019; Cust et al., 2019). The growing availability of event, tracking, and positional data has also enabled increasingly sophisticated analyses of tactical behavior and competitive performance (Goes et al., 2021). In football, for example, AI-based systems can integrate large volumes of match events to produce multidimensional and position-sensitive evaluations of individual players (Pappalardo et al., 2019). Together, these developments make AI a plausible source not only for collecting and processing performance data but also for interpreting and evaluating what those data reveal about an athlete.

The technical capacity to produce an athlete evaluation, however, does not guarantee that an AI-generated evaluation will be perceived in the same way as one attributed to a human expert (Longoni et al., 2019). People use information about a source to infer how a judgment was reached and whether the source possesses the abilities required for the task. Although some research has found that individuals may favor algorithmic advice over human advice, other studies show that responses to algorithms vary considerably according to the type of judgment involved and the role assigned to the algorithm (Castelo et al., 2019; Logg et al., 2019). Merely identifying an evaluation as algorithmic rather than human can also change how people interpret and respond to the resulting decision (Yalcin et al., 2022). Thus, the perceived value of sport performance information may depend not only on its substantive content but also on whether the evaluator is presented as an AI system or a human analyst.

Athlete performance assessment provides a particularly relevant setting in which to examine this source effect because competitive performance is not only objectively measurable but also inherently context dependent (MacKenzie and Cushion, 2013; Sarmento et al., 2014). Technical and physical indicators can provide valuable information about athlete performance, but their interpretation may vary according to playing position, tactical responsibilities, opponent quality, match status, and broader competitive conditions (Trewin et al., 2017; Yi et al., 2020). Performance indicators should therefore be interpreted in relation to the situational and interactional context in which they are produced rather than treated as isolated measures of ability (Sarmento et al., 2014). Athlete evaluation consequently requires the analysis of numerical indicators as well as the interpretation and integration of tactical, contextual, and athlete-specific information (Plessner and Haar, 2006).

Task-dependent algorithm aversion provides a framework for explaining how people may respond to AI-based athlete evaluations. Reliance on algorithms depends partly on how the task is perceived, with algorithms receiving less trust for subjective tasks and more favorable responses for objective and computational tasks (Castelo et al., 2019). This tendency may be reinforced when people believe that AI cannot adequately account for an individual’s unique characteristics and circumstances (Longoni et al., 2019; Yalcin et al., 2022). Although AI systems may appear well suited to processing extensive statistical and tracking data, interpreting athlete performance may still be viewed as requiring sport-specific experience, tactical insight, and the integration of contextual information (Plessner and Haar, 2006). Accordingly, individuals may regard professional analysts as more appropriate than AI systems for evaluating an athlete’s performance, a possibility also suggested by research showing that perceived human expertise shapes responses to AI-generated recommendations in sport contexts (Buechner et al., 2025).

Despite the growing use of AI in sport, existing research has largely focused on the technical development, accuracy, and predictive performance of AI-based applications. Much of this literature has prioritized whether algorithmic systems can successfully predict injuries, recognize movements, analyze tactics, or generate performance evaluations rather than how stakeholders perceive the evaluations these systems produce (Claudino et al., 2019; Cust et al., 2019; Pappalardo et al., 2019). At the same time, transparency, explainability, accountability, and trust have emerged as important concerns surrounding the implementation of algorithmic systems in sport (Kim et al., 2025). Nevertheless, comparatively little is known about whether an athlete performance evaluation is perceived differently when attributed to AI rather than human expertise, despite growing interest in responses to AI recommendations and sport-related predictions (Buechner et al., 2025; Kornowicz and Thommes, 2025). Examining evaluations attributed to AI-based systems and professional human analysts may therefore provide a clearer understanding of how source attribution shapes perceptions of athlete performance information.

The purpose of this study is to examine whether an athlete performance report is evaluated differently when attributed to an AI-based analysis system or professional human analysts. Guided by task-dependent algorithm aversion, the study investigates how the attributed source of the report shapes perceptions of its credibility, its diagnostic value for understanding athlete performance, and the extent to which the evaluation appears to consider the athlete’s unique characteristics. By applying this theoretical perspective to athlete performance assessment, the study examines algorithmic judgment in a context that combines quantitative performance information with contextual and individualized interpretation. This study extends research on algorithm aversion to athlete performance assessment and provides insight into how the growing use of AI may shape perceptions of expertise in sport.

## 2 Literature review and hypotheses development

### 2.1 Task-dependent algorithm aversion and athlete performance evaluation

Research on algorithmic judgment has shown that people do not respond uniformly to advice or evaluations produced by algorithms. Individuals may reject algorithmic judgments after observing even minor errors and may penalize such errors more strongly than comparable mistakes made by human decision makers (Dietvorst et al., 2015; Prahl and Van Swol, 2017). In other settings, however, algorithmic advice may be accepted or preferred, particularly when the task is structured and the algorithm is perceived as capable of producing accurate and consistent judgments. A review of task-specific algorithm acceptance similarly indicates that reactions to algorithmic advice vary substantially across decision contexts rather than reflecting a general preference for either algorithms or humans (Kaufmann et al., 2023).

Task-dependent algorithm aversion explains this variation by focusing on the perceived fit between the evaluator and the task. People tend to view algorithms as more appropriate for objective tasks involving calculation, prediction, and rule-based processing, whereas human judgment is generally preferred for tasks perceived as subjective or dependent on intuition and personal understanding (Castelo et al., 2019). For example, people have shown reluctance to rely on algorithms for preference-based recommendations, even when algorithmic recommendations outperform those provided by other individuals (Yeomans et al., 2019). These findings suggest that evaluations of AI depend not only on its actual capabilities but also on whether those capabilities are perceived to match the demands of the task.

Athlete performance evaluation combines extensive quantitative information with judgments about what that information means within a particular sporting context. Although AI can efficiently process statistical and tracking data, professional expertise in sport is associated with the ability to recognize meaningful patterns, identify relevant cues, and interpret information in relation to domain-specific conditions (Mann et al., 2007; Williams and Ford, 2008). Evaluating an athlete may therefore be perceived as requiring contextual and experiential judgment that is more strongly associated with professional human analysts. This perceived correspondence between human expertise and the requirements of athlete assessment provides the basis for expecting different responses to otherwise comparable AI- and human-attributed reports.

At the same time, athlete performance evaluation also contains a strongly quantitative and data-intensive component. AI-based systems may therefore be perceived as well suited to systematically processing extensive performance information, applying consistent criteria, and identifying patterns across multiple indicators. Thus, athlete performance evaluation contains competing source-fit cues: AI may be favored when recipients emphasize systematic, data-intensive processing, whereas human analysts may be favored when they emphasize contextual and individualized interpretation. The hypotheses developed below reflect the expectation that the contextual demands of athlete evaluation would be more salient in the present setting, while recognizing that source effects may differ across evaluative dimensions.

### 2.2 Message credibility

Message credibility refers to the degree to which a message is perceived as accurate, authentic, and believable (Appelman and Sundar, 2016). Although message credibility concerns the evaluation of content rather than the source itself, recipients often use available source information as a cue when they cannot independently verify every claim contained in a message (Foy et al., 2017; Slater and Rouner, 1996). The identity of the reported author can therefore shape assumptions about how the information was produced and whether it deserves belief (Sundar, 2008).

Research on automated content demonstrates that perceived machine authorship can influence credibility judgments even when recipients encounter comparable substantive information (Waddell, 2018). More recent evidence indicates that labeling content as AI-generated can reduce its perceived accuracy regardless of whether the information is true or whether it was actually produced by AI (Altay and Gilardi, 2024). These findings suggest that source attribution can influence evaluations of a message independently of its substantive quality. Human attribution may therefore provide a favorable credibility cue when producing the message is perceived to require expertise and informed judgment.

This logic is applicable to athlete performance reports because their credibility depends on more than the accurate presentation of statistics. Readers must also believe that the report offers a valid interpretation of the athlete’s actions, responsibilities, and overall contribution. Professional human analysts may be assumed to possess the sport knowledge and practical judgment needed to produce such an interpretation, whereas an AI system may be associated more narrowly with data processing. At the same time, the structured and quantitative nature of performance reports may activate expectations that AI can apply evaluative criteria systematically and consistently, which could favor AI in credibility judgments. Nevertheless, because athlete performance evaluation also requires informed interpretation of the athlete’s actions, responsibilities, and competitive context, we expected the human-expertise cue to be more salient for message credibility. Accordingly, the following hypothesis is proposed:

*H1*: An athlete performance report attributed to professional human analysts will be perceived as more credible than a report attributed to an AI-based analysis system.

### 2.3 Perceived diagnosticity

Perceived diagnosticity refers to the extent to which information helps an individual understand and evaluate a target (Jiang and Benbasat, 2004). Information is considered diagnostic when it clarifies the meaning of available evidence and supports the formation of an overall judgment. Importantly, perceived diagnosticity concerns the recipient’s subjective assessment of whether information is useful for understanding and evaluating a target, rather than the objective accuracy of that evaluation relative to an external benchmark. In this respect, perceived diagnosticity is also conceptually distinct from message credibility, which concerns whether the message itself is perceived as accurate, authentic, and believable. Research on information adoption indicates that people are more likely to regard information as useful when it is well reasoned and provided by a credible and knowledgeable source (Sussman and Siegal, 2003). Similarly, the relevance, factuality, currency, and source credibility of information can influence its perceived diagnostic value for evaluation and decision-making (Filieri, 2015; Filieri et al., 2018).

For an athlete performance report, diagnosticity concerns whether the report helps readers move beyond isolated statistics and develop a clearer understanding of the athlete’s performance. The same indicators may have different implications depending on the athlete’s position, assigned role, tactical system, and competitive circumstances. Accordingly, meaningful performance analysis requires the identification and interpretation of relationships among multiple forms of technical, positional, and tactical information (Rein and Memmert, 2016). A report is therefore likely to appear more diagnostic when its source is believed to understand which information is relevant and how that information should be integrated.

Professional human analysts may be perceived as possessing the domain knowledge needed to distinguish meaningful performance indicators from less informative ones. Their evaluations may consequently be viewed as offering interpretation rather than simply summarizing observable data. In contrast, an AI-attributed report may be perceived as reflecting the systematic processing of available indicators without the same capacity to determine their broader sporting significance. Conversely, when a performance report is perceived as highly data intensive, AI may be viewed as capable of systematically integrating multiple indicators and identifying relevant patterns, which could enhance its perceived diagnostic value. Nevertheless, because evaluating athlete performance also requires interpreting the broader sporting significance of those indicators, we expected the human-attributed report to provide greater perceived diagnostic value. This reasoning leads to the following hypothesis:

*H2*: An athlete performance report attributed to professional human analysts will have greater perceived diagnosticity than a report attributed to an AI-based analysis system.

### 2.4 Perceived consideration of athlete uniqueness

Perceived consideration of athlete uniqueness refers to the extent to which an evaluator is believed to recognize and incorporate the distinctive characteristics and circumstances of an individual case. This consideration is particularly relevant to algorithmic evaluation because people may perceive AI systems as relying on standardized procedures that inadequately reflect individual differences. Longoni et al. (2019) described this concern as uniqueness neglect, whereby resistance to AI arises from the belief that algorithms cannot sufficiently account for an individual’s unique characteristics and circumstances. This perception does not necessarily indicate that algorithms are technically incapable of personalization; rather, it reflects the belief that algorithmic evaluations may reduce individuals to standardized inputs and overlook qualities that are difficult to quantify.

Research across decision, recommendation, and service contexts supports the importance of perceived individualization in responses to AI. Algorithmic decision makers may be viewed as less responsive than humans to the distinctive features of an individual case (Yalcin et al., 2022). Preferences for AI-based recommendations may also weaken when an evaluation requires matching information to a person’s unique preferences, as such individualized understanding is often associated more strongly with human judgment (Longoni and Cian, 2022). Perceived uniqueness neglect has similarly been linked to consumer resistance toward AI services beyond the original medical context (Mou et al., 2023). Together, these findings suggest that evaluations attributed to humans may be perceived as more responsive to individual characteristics and circumstances.

This distinction is especially relevant to athlete performance assessment because athletes differ in their positional responsibilities, tactical functions, playing styles, physical attributes, and contributions to team performance. Even athletes occupying the same position may be assigned different responsibilities according to team strategy and competitive circumstances. Professional analysts may therefore be perceived as better able to recognize these differences and incorporate them into an overall performance evaluation. In contrast, an AI-based system may be viewed as relying more heavily on standardized indicators and general evaluation criteria. Accordingly, the following hypothesis is proposed:

*H3*: An athlete performance report attributed to professional human analysts will be perceived as showing greater consideration of the athlete’s unique characteristics than a report attributed to an AI-based analysis system.

## 3 Methods

### 3.1 Research design and participants

This study employed a one-factor, two-condition, between-subjects online experimental design. The independent variable was the attributed source of an athlete performance report, with two conditions: an AI-based analysis system and professional human analysts. Participants were randomly assigned to one of the two conditions.

An *a priori* power analysis conducted using G*Power 3.1 indicated that a minimum sample of 128 participants was required to detect a medium effect (*f* = 0.25) with an alpha level of 0.05 and statistical power of 0.80. The final sample exceeded this requirement. The study received approval from the institutional review board, and all participants provided informed consent before participating in the experiment.

Participants were recruited through Prolific from adults residing in the United Kingdom who identified as football fans. Eligibility was confirmed by correctly identifying Kevin De Bruyne from a photograph. A total of 235 participants were initially recruited, of whom 15 were excluded because they failed the manipulation check by incorrectly identifying whether the performance report had been produced by AI or human analysts. The final sample therefore consisted of 220 participants, with 110 assigned to the AI condition and 110 assigned to the human analyst condition. Participants ranged in age from 19 to 45 years (*M* = 29.44, *SD* = 6.09). The sample included 129 men (58.6%), 89 women (40.5%), and two participants who selected another gender identity or preferred not to disclose their gender (0.9%).

### 3.2 Experimental stimulus and source manipulation

The experimental stimulus was a standardized match performance profile evaluating professional football player Kevin De Bruyne. The profile presented quantified ratings of 10 performance attributes, including vision, passing, creativity, ball progression, chance creation, positioning, decision-making, ball retention, defensive contribution, and physical duels. The ratings were displayed using numerical scores, horizontal rating bars, and a radar chart. The profile also included an overall match-impact rating, a positional heat map, and brief summaries of the athlete’s primary strengths and limitations.

The substantive content, length, organization, and visual presentation of the report were identical across the two experimental conditions. Only the attributed source was manipulated. In the AI condition, the report was labeled “Source of Analysis: AI-Based Football Performance Analysis System,” followed by the statement “The following report was produced by an AI-based football performance analysis system.” In the human analyst condition, the corresponding wording was “Source of Analysis: Professional Football Performance Analysts,” followed by the statement “The following report was produced by professional football performance analysts.” These source labels and accompanying statements were the only textual elements that differed between conditions; all other substantive and visual elements of the stimulus were identical.

### 3.3 Procedure

The experiment was administered through an online survey platform. After providing informed consent, participants completed a measure of their familiarity with Kevin De Bruyne. They were then assigned to either the AI-based analysis system condition or the professional human analyst condition in a 1:1 ratio using the online survey platform’s built-in randomization function. Participants then viewed a brief description identifying the source responsible for producing the report. The attributed source was also displayed in the heading immediately above the performance report.

Participants were instructed to read the report carefully and evaluate it based on the information provided. After reviewing the report, they completed measures of message credibility, perceived diagnosticity, and perceived consideration of athlete uniqueness. The three measurement blocks were presented in randomized order to reduce potential order effects. Participants then completed the manipulation check.

An instructional attention-check item was included to identify inattentive responses, asking participants to select a specified response option. Responses were screened for incompleteness, duplicate participation, failed attention checks, and implausibly short completion times. Participants who incorrectly identified the attributed source were excluded from the final analytic sample. This exclusion criterion was applied because the experimental manipulation concerned the attributed source of the report; participants who did not correctly recognize their assigned source could not be assumed to have received the intended source-attribution manipulation. No additional participants were excluded based on the remaining screening criteria. At the end of the study, participants were informed that the attributed source had been experimentally manipulated and that the report had been developed for research purposes.

### 3.4 Measures

As a source-recognition check, participants identified whether the report had been produced by an AI-based analysis system or professional human analysts. Familiarity with Kevin De Bruyne was measured using three seven-point semantic differential items adapted from Machleit et al. (1993), including unfamiliar/familiar, inexperienced/experienced, and not knowledgeable/knowledgeable. The averaged score was included as a covariate in the main analysis.

Message credibility was measured using three items from Appelman and Sundar (2016). Participants rated the extent to which the report was *accurate*, *authentic*, and *believable*. Perceived diagnosticity was measured using two items adapted from Uhm et al. (2022): “This report was diagnostic for evaluating the athlete’s performance” and “This report provided information that helped me judge the athlete’s performance.” Accordingly, these items assessed participants’ perceived usefulness of the report for forming an evaluation of the athlete rather than their ability to verify the report against an objective account of the actual match. Perceived consideration of athlete uniqueness was measured using three items adapted from Yalcin et al. (2022). Example items assessed whether the report recognized the athlete’s unique performance characteristics and considered the unique aspects of the athlete’s performance. Items were averaged within each construct, with higher scores indicating higher levels of the respective variable. All continuous measures used seven-point response scales.

### 3.5 Data analysis

The data were analyzed using IBM SPSS Statistics 31.0. Before the main analyses, the data were screened for incomplete responses, duplicate participation, failed attention checks, and implausibly short completion times. Means, standard deviations, skewness, kurtosis, Cronbach’s alpha coefficients, and Pearson correlations were calculated for the study variables.

The effectiveness of the source manipulation was assessed based on whether participants correctly identified the source assigned to their experimental condition. Before conducting the multivariate analysis of covariance (MANCOVA), the homogeneity of variance–covariance matrices, homogeneity of variance, and homogeneity of regression slopes were examined. Box’s *M* test, Levene’s tests, and the interaction between attributed source and familiarity were used to assess these assumptions, respectively. Pillai’s trace was used as the primary multivariate statistic. When the multivariate effect was significant, follow-up univariate analyses of covariance were conducted for the three dependent variables. Adjusted means, standard errors, and partial eta squared values were reported, and statistical significance was evaluated at *α* = 0.05. To examine whether exclusion based on the source-recognition check materially influenced the results, the primary MANCOVA and follow-up ANCOVAs were repeated as a sensitivity analysis using all 235 initially recruited participants.

## 4 Results

### 4.1 Preliminary analyses and manipulation check

Descriptive statistics, internal consistency coefficients, and correlations among the study variables are presented in Table 1. Skewness values ranged from −0.241 to 0.068, and kurtosis values ranged from −0.588 to 0.275, indicating no substantial departure from univariate normality. Cronbach’s alpha coefficients ranged from 0.806 to 0.830, demonstrating acceptable internal consistency. Correlations among the study variables ranged from 0.132 to 0.430, suggesting that multicollinearity was not a concern.

**Table 1:** Descriptive statistics, reliability, and correlations among study variables*.*

Variable	*M*	*SD*	Skewness	Kurtosis	*α*	1	2	3	4
1. Familiarity	4.956	0.895	−0.241	−0.308	0.830	—			
2. Message credibility	4.845	0.797	0.068	0.275	0.822	0.141*	—		
3. Perceived diagnosticity	4.830	0.806	−0.191	−0.197	0.806	0.132	0.430***	—	
4. Consideration of uniqueness	4.544	0.875	−0.032	−0.588	0.819	0.160	0.350***	0.297***	—

* *p* < 0.05. *** *p* < 0.001.

Of the 235 initially recruited participants, 220 (93.6%) correctly identified the attributed source. The 15 participants who failed the source-recognition check were excluded, indicating that all participants in the final analytic sample correctly recognized their assigned source. The assumptions examined for MANCOVA were also satisfied. Box’s *M* test was nonsignificant, Box’s *M* = 7.687, *p* = 0.271. Levene’s tests were nonsignificant for message credibility, perceived diagnosticity, and consideration of athlete uniqueness, with *p*-values ranging from 0.238 to 0.528. In addition, the interaction between attributed source and familiarity was not significant, Pillai’s *V* = 0.011, *F*(3, 214) = 0.824, *p* = 0.482, supporting the homogeneity-of-regression-slopes assumption.

### 4.2 Hypothesis tests

A one-way MANCOVA was conducted to examine the effect of attributed source on message credibility, perceived diagnosticity, and perceived consideration of athlete uniqueness while controlling for familiarity with Kevin De Bruyne. The multivariate effect of attributed source was significant, Pillai’s *V* = 0.309, *F*(3, 215) = 32.061, *p* < 0.001, partial *η*^2^ = 0.309. Familiarity with Kevin De Bruyne also had a significant multivariate association with the combined dependent variables, Pillai’s *V* = 0.038, *F*(3, 215) = 2.793, *p* = 0.041.

As shown in Table 2, the follow-up ANCOVAs revealed different source effects across the three evaluation dimensions. Contrary to H1, message credibility was significantly higher in the AI condition than in the human analyst condition, *F*(1, 217) = 11.478, *p* = 0.001, partial *η*^2^ = 0.050. Contrary to H2, perceived diagnosticity was also significantly higher in the AI condition, *F*(1, 217) = 21.795, *p* < 0.001, partial *η*^2^ = 0.091. In contrast, the human-attributed report was perceived as showing greater consideration of athlete uniqueness, *F*(1, 217) = 23.292, *p* < 0.001, partial *η*^2^ = 0.097, supporting H3. Thus, H1 and H2 were not supported because the significant effects emerged in the direction opposite to that hypothesized, whereas H3 was supported.

**Table 2:** Adjusted means and ANCOVA results by attributed source.

Outcome	Adjusted AI *M* (SE)	Adjusted Human *M* (*SE*)	*F*	*p*	Partial *η*^2^
Message credibility	5.022 (0.074)	4.669 (0.074)	11.478	0.001	0.050
Perceived diagnosticity	5.071 (0.073)	4.589 (0.073)	21.795	<0.001	0.091
Consideration of uniqueness	4.276 (0.079)	4.812 (0.079)	23.292	<0.001	0.097

Adjusted means were estimated while controlling for familiarity. All univariate tests used df = 1, 217. AI, AI-based analysis system; human, professional human analysts.

As a sensitivity analysis, the primary analyses were repeated using all 235 initially recruited participants, including the 15 participants who failed the source-recognition check. The substantive pattern of results remained unchanged. The multivariate effect of attributed source remained significant, Pillai’s *V* = 0.310, *F*(3, 230) = 34.515, *p* < 0.001, partial *η*^2^ = 0.310. Follow-up ANCOVAs likewise showed higher message credibility in the AI condition, *F*(1, 232) = 13.309, *p* < 0.001, partial *η*^2^ = 0.054, and higher perceived diagnosticity in the AI condition, *F*(1, 232) = 23.839, *p* < 0.001, partial *η*^2^ = 0.093. Perceived consideration of athlete uniqueness remained significantly higher in the human analyst condition, *F*(1, 232) = 24.561, *p* < 0.001, partial *η*^2^ = 0.096. Thus, the direction and substantive interpretation of the findings were not dependent on excluding participants who failed the source-recognition check.

## 5 Discussion

### 5.1 General discussion

The current study examined whether the attributed source of an identical athlete performance report shaped how the report was evaluated. The findings revealed a differentiated source-attribution pattern. Contrary to H1 and H2, AI attribution increased message credibility and perceived diagnosticity, whereas, consistent with H3, human attribution increased perceived consideration of athlete uniqueness. These findings indicate that responses to AI-based athlete evaluation cannot be characterized as uniformly favorable or unfavorable. Instead, the same source cue produced different evaluations depending on whether participants considered the report’s analytical credibility and usefulness or its sensitivity to the athlete’s individual characteristics. The reversal of H1 and H2 suggests that, in the present stimulus, the report’s structured and quantitative features may have made computational source-fit cues more salient for credibility and diagnosticity judgments than the contextual-expertise cues emphasized in the original hypotheses. At the same time, the corresponding univariate effect sizes (partial *η*^2^ = 0.050–0.097) should be interpreted as dimension-specific source-attribution effects within the present experimental context rather than as large or universal preferences for either AI or human analysts.

First, contrary to H1, the report attributed to the AI-based analysis system was perceived as more credible than the identical report attributed to professional human analysts. This finding suggests that AI attribution did not create a general credibility penalty in the context of athlete performance evaluation. Participants may instead have inferred that an AI-based system could process performance indicators systematically, apply consistent evaluative criteria, and reach conclusions with less susceptibility to subjective bias. Comparable patterns have emerged in automated journalism, where automated sports articles were sometimes perceived as more credible than human-written articles despite containing substantively similar information (Wölker and Powell, 2021). Recent research on fact-checking also suggests that AI can be viewed favorably for relatively structured tasks because it is associated with objectivity and freedom from personal bias (Kim and Lee, 2026). In the present study, the quantitative and performance-oriented appearance of the report may therefore have activated expectations that AI was especially capable of producing an accurate and evidence-based evaluation.

This result should nevertheless be interpreted as a perception of credibility rather than evidence that the AI-attributed report was objectively more accurate. The report was identical across conditions, and participants were not given information about the amount, quality, or diversity of the data used by either source. Their evaluations therefore appear to reflect assumptions activated by the AI label rather than verified differences in analytical quality. This distinction is important because research on AI authorship has produced heterogeneous credibility effects. A recent meta-analysis found a small average credibility penalty for AI-labeled news but also substantial variation across studies, including null and reversed effects depending on the communication context (Kim et al., 2026). The present result adds to this mixed evidence by suggesting that AI attribution may enhance message credibility when the task appears data intensive and computationally structured. Thus, credibility responses to AI may depend less on a stable preference for humans or machines than on the capabilities that recipients associate with each source in a particular evaluative setting.

Second, contrary to H2, the AI-attributed report was perceived as more diagnostic for understanding and evaluating the athlete’s performance. This unexpected result indicates that source attribution affected not only general trust in the report but also perceptions of how useful the report was for forming a substantive judgment. Participants may have assumed that an AI system could integrate multiple performance indicators, identify patterns across extensive match data, and distinguish meaningful information from less relevant observations. Such assumptions could make the same report appear more analytically informative when presented as the product of AI. Recent evidence similarly shows that knowledge workers may respond more favorably to generative AI advice than to human expert advice during complex tasks, particularly when they trust AI’s technical capability (Amrollahi et al., 2026). Research also indicates that people increasingly rely on algorithmic advice as tasks become more difficult, suggesting that computational sources may be perceived as especially useful when the information-processing demands of a judgment are high (Bogert et al., 2021).

More importantly, the finding demonstrates that perceived diagnosticity is not determined solely by the substantive information contained in a message. Although both groups received the same ability ratings, visualizations, and evaluative summaries, the AI label may have changed participants’ beliefs about how those conclusions had been generated. The report may consequently have appeared to represent a more comprehensive or sophisticated analytical process, even though no additional information was actually provided. This interpretation is consistent with evidence that algorithmic advice can increase recipients’ confidence in their judgments independently of whether it improves objective performance. In a word-association experiment, participants became more confident and changed their responses more frequently after receiving algorithmic rather than human advice, although their final answers were less likely to be correct (Bogert et al., 2022). Accordingly, the higher perceived diagnosticity observed here should not be interpreted as evidence of greater objective diagnostic accuracy or closer correspondence with the athlete’s actual match performance. Rather, it indicates that AI attribution increased the perceived informational value of otherwise identical content for understanding and evaluating the athlete.

Third, consistent with H3, the report attributed to professional human analysts was perceived as giving greater consideration to the athlete’s unique characteristics. This result contrasts with the advantages assigned to AI for credibility and diagnosticity and shows that participants differentiated between systematic information processing and individualized understanding. Athlete performance cannot be interpreted entirely through standardized indicators because the meaning of an action may depend on the athlete’s playing style, tactical responsibilities, interactions with teammates, and the specific competitive situation. Participants may therefore have assumed that professional human analysts could draw on experiential knowledge and contextual insight to recognize features of the athlete that could not be fully represented through standardized data. Similar tensions have been observed in AI-based personnel evaluation. AI interviews can generate stronger fairness expectations while simultaneously producing greater concerns that the system will neglect an applicant’s distinctive competencies and qualities (Wang et al., 2025). The human-attributed report may therefore have benefited from expectations of individualized and context-sensitive judgment.

At the same time, the finding should not be taken to mean that AI systems are inherently incapable of individualized evaluation. Because the report itself was identical, the difference again reflects participants’ beliefs about the source rather than demonstrated differences in personalization. Research on personalized services indicates that AI-generated solutions may be perceived as less unique than human-generated solutions, partly because users assume that the same algorithm serves a large number of individuals (Hou et al., 2026). However, experimental evidence also shows that AI agents can benefit when they explicitly incorporate individualized social and case-specific information, suggesting that perceptions of impersonalization can be mitigated through appropriate system design (Chen et al., 2024). The present result therefore identifies a perceived limitation rather than a fixed technological limitation. AI-based athlete evaluation may be seen as more responsive to athlete uniqueness when systems clearly communicate how positional responsibilities, tactical context, playing style, and individual performance history have been incorporated into the evaluation.

### 5.2 Theoretical and practical implications

The current study advances research on algorithmic judgment by demonstrating that algorithm appreciation and algorithm aversion can coexist within the same evaluative task. Previous research has often examined whether people prefer algorithms or humans across different types of tasks. The present findings show that such preferences may also vary across the evaluative dimensions embedded within a single task. AI attribution enhanced message credibility and perceived diagnosticity, whereas human attribution increased the perceived consideration of athlete uniqueness. Athlete performance evaluation therefore did not elicit a uniformly positive or negative response toward AI. Instead, participants appeared to associate AI with systematic information processing and human analysts with individualized contextual understanding. This dimension-specific pattern supports recent arguments that judgments of computer agents may be more variable and differentiated than a simple algorithm-aversion account implies (Buder et al., 2024). The findings consequently suggest that responses to algorithmic evaluation should be conceptualized as a multidimensional profile of perceived capabilities rather than as a general preference for either AI or human judgment. These perceived capability differences should not be interpreted as evidence of objective differences in the underlying analytical performance of AI and human analysts.

The findings of this study extend source-attribution research by showing that a source label can change the perceived functional value of otherwise identical information. Participants received the same performance indicators and narrative evaluation, yet AI attribution increased the extent to which the report appeared credible and useful for understanding the athlete. This suggests that recipients do not evaluate report content independently of their beliefs about how it was produced. Instead, the source label may activate a mental model of the evaluator, including assumptions about its information-processing capacity, consistency, expertise, and sensitivity to contextual detail. Research on AI-assisted decision-making similarly emphasizes that people’s mental models of AI shape how they interpret and use algorithmic information (Steyvers and Kumar, 2024), while experimental work indicates that algorithmic advice may prompt cognitive processing that differs from responses to human advice (van Maanen et al., 2026). The present study therefore identifies the inferred production process as a potentially important mechanism linking source attribution to message evaluation. Future research could directly test whether perceived objectivity, analytical comprehensiveness, or contextual sensitivity mediates these differentiated source effects.

From a practical perspective, the present findings concern how source attribution shapes perceptions of athlete performance information rather than the objective division of analytical tasks between AI and human analysts. Because the report content was held constant across conditions, the results do not establish that AI is objectively superior in data integration or systematic processing, nor that human analysts are objectively superior in contextual or individualized interpretation. Instead, they show that identical performance information may be evaluated differently depending on whether it is attributed to an AI system or professional human analysts. For sport organizations, this finding highlights the importance of source disclosure and the communication surrounding AI-supported performance reports. Clearly communicating how a report was produced, what information informed the evaluation, and whether human review was involved may help users interpret the report without inferring capabilities that were not directly demonstrated. Direct comparisons of AI-generated, human-generated, and human–AI collaborative reports are therefore needed before specific recommendations can be made regarding the operational allocation of analytical responsibilities. These practical implications should also be interpreted cautiously because the present participants were football fans rather than professional sport decision makers; their applicability to organizational practice should therefore be established through replication with coaches, scouts, performance analysts, and other sport professionals.

The findings also have implications for how AI-supported performance reports are presented to athletes, coaches, media professionals, and fans. Because AI attribution alone increased credibility and diagnosticity in the present experiment, organizations should be careful not to allow favorable assumptions about AI’s analytical capacity to develop into uncritical reliance. Reports should clearly identify the data sources, time periods, indicators, and assumptions used in producing the evaluation, while also communicating uncertainty and the limits of the system. At the same time, concerns about uniqueness neglect may be reduced by showing how athlete-specific information—such as playing position, tactical role, competitive context, and longitudinal performance patterns—was incorporated into the analysis. A visible statement that the report was reviewed or contextualized by a qualified human analyst may further clarify the division of responsibility. Importantly, explanation should be designed to calibrate trust rather than merely increase it, as empirical evidence indicates that some explanation formats can unintentionally encourage excessive reliance on AI recommendations (Naiseh et al., 2023). Sport organizations should therefore test different reporting formats with coaches, athletes, analysts, and general audiences before adopting them in consequential performance decisions.

### 5.3 Limitations and future research

This study has several limitations. First, the experimental context was restricted to a single performance report concerning one highly recognizable professional football player. Kevin De Bruyne’s reputation, playing position, and established image as an elite and tactically intelligent midfielder may have influenced how participants interpreted the report and its attributed source. The findings may therefore not generalize to less familiar athletes, different playing positions, other levels of competition, or sports in which performance is assessed through different combinations of objective and subjective information. Future studies should replicate the experiment using multiple athletes, playing positions, competition levels, matches, sports, and report formats, including performance reports in which the available information is more ambiguous or open to interpretation. Accordingly, the present findings should be interpreted as context-specific source-attribution effects rather than as evidence that the same AI–human evaluation pattern will necessarily emerge across athletes or sport settings. Comparing highly quantitative reports with more interpretive reports would also help determine whether AI attribution becomes more advantageous as the evaluation appears increasingly data driven, whereas human attribution becomes more favorable as greater contextual or interpersonal judgment is required.

Second, the study manipulated only the attributed source while holding the report content constant. This design was intentionally used to isolate the independent effect of source attribution, but it did not compare reports actually generated by AI systems and professional analysts and therefore cannot establish objective differences in their analytical capabilities. The results therefore reflect participants’ assumptions about AI and human evaluators rather than demonstrated differences in their analytical accuracy, comprehensiveness, or capacity for personalization. In addition, the dependent variables captured perceived credibility, diagnosticity, and consideration of athlete uniqueness rather than objective judgment quality, behavioral reliance, or actual adoption of the report’s recommendations. Moreover, because participants were not shown the match itself or provided with an external performance benchmark, the present study cannot determine whether the report objectively corresponded with the athlete’s actual performance in that specific match. Future research could compare authentic AI-generated, human-generated, and AI–human collaborative reports while assessing decision accuracy, willingness to rely on the evaluation, and subsequent behavioral choices. Future research should directly test whether perceived objectivity, analytical comprehensiveness, contextual sensitivity, and uniqueness neglect mediate the effects of source attribution on credibility, diagnosticity, and perceived individualized consideration. Such analyses would help identify the psychological mechanisms underlying the differentiated responses to AI- and human-attributed performance reports.

The participant sample presents an important boundary condition. Although football fans are relevant audiences for athlete performance information, the present findings should be interpreted primarily as reflecting fan evaluations rather than professional sport decision-making. Coaches, scouts, performance analysts, and sport administrators typically possess greater domain expertise and may evaluate AI- and human-attributed reports differently, particularly when such reports inform consequential decisions. The online experimental setting also does not reproduce the time pressure, accountability, and organizational constraints that characterize professional performance evaluation. Decision stakes may therefore represent an important boundary condition: source-attribution effects observed in a relatively low-stakes fan evaluation may differ from those arising in scouting, player-selection, or tactical decisions for which professional decision makers are accountable. Future research should test whether the present pattern replicates among sport professionals and in field settings. Because AI literacy and prior experience with AI-based analytics were not directly measured in the present study, their moderating roles could not be examined. Individual differences such as AI literacy, prior experience with AI or sport analytics, trust in technology, football expertise, and general attitudes toward AI may also moderate responses to source attribution and should be examined systematically.

## Data Availability

The raw data supporting the conclusions of this article will be made available by the authors, without undue reservation.
